# SAOS-2 Osteosarcoma Cells Bind Fibroblasts via ICAM-1 and This Is Increased by Tumour Necrosis Factor-α

**DOI:** 10.1371/journal.pone.0101202

**Published:** 2014-06-30

**Authors:** Manu S. David, Elizabeth Kelly, Ivan Cheung, Munira Xaymardan, Malcolm A. S. Moore, Hans Zoellner

**Affiliations:** 1 The Cellular and Molecular Pathology Research Unit, Department of Oral Pathology and Oral Medicine, Faculty of Dentistry, The University of Sydney, Westmead Centre for Oral Health, Westmead Hospital, Westmead, New South Wales, Australia; 2 Memorial Sloan-Kettering Cancer Center, New York, New York, United States of America; IISER-TVM, India

## Abstract

We recently reported exchange of membrane and cytoplasmic markers between SAOS-2 osteosarcoma cells and human gingival fibroblasts (h-GF) without comparable exchange of nuclear markers, while similar h-GF exchange was seen for melanoma and ovarian carcinoma cells. This process of “cellular sipping” changes phenotype such that cells sharing markers of both SAOS-2 and h-GF have morphology intermediate to that of either cell population cultured alone, evidencing increased tumour cell diversity without genetic change. TNF-α increases cellular sipping between h-GF and SAOS-2, and we here study binding of SAOS-2 to TNF-α treated h-GF to determine if increased cellular sipping can be accounted for by cytokine stimulated SAOS-2 binding. More SAOS-2 bound h-GF pe-seeded wells than culture plastic alone (p<0.001), and this was increased by h-GF pre-treatment with TNF-α (p<0.001). TNF-α stimulated binding was dose dependent and maximal at 1.16nM (p<0.05) with no activity below 0.006 nM. SAOS-2 binding to h-GF was independent of serum, while the lipopolysaccharide antagonist Polymyxin B did not affect results, and TNF-α activity was lost on boiling. h-GF binding of SAOS-2 started to increase after 30min TNF-α stimulation and was maximal by 1.5hr pre-treatment (p<0.001). h-GF retained maximal binding up to 6hrs after TNF-α stimulation, but this was lost by 18hrs (p<0.001). FACS analysis demonstrated increased ICAM-1 consistent with the time course of SAOS-2 binding, while antibody against ICAM-1 inhibited SAOS-2 adhesion (p<0.04). Pre-treating SAOS-2 with TNF-α reduced h-GF binding to background levels (p<0.003), and this opposite effect to h-GF cytokine stimulation suggests that the history of cytokine exposure of malignant cells migrating across different microenvironments can influence subsequent interactions with fibroblasts. Since cytokine stimulated binding was comparable in magnitude to earlier reported TNF-α stimulated cellular sipping, we conclude that TNF-α stimulated cellular sipping likely reflects increased SAOS-2 binding as opposed to enhanced exchange mechanisms.

## Introduction

Malignant neoplasms arise from acquisition of somatic mutations during initiation, expansion of clones of initiated cells through the action of proliferative signals in promotion, and emergence of increasingly malignant sub-clones to result in disease progression [1], [2]. While it is convenient and informative to study isolated neoplastic parenchymal cells cultured out of malignancies, there is increasing evidence that complex interactions between malignant parenchymal cells and supporting stromal cells play an important role in cancer [3]–[8].

Of particular relevance to the current work is our recent paper describing the exchange of membrane and cytoplasm between cultured parenchymal malignant cells and human gingival fibroblasts (h-GF), a process we have termed ‘cellular sipping’ [9]. In that study we observed the exchange of separate membrane and cytoplastmic fluorescent markers in the absence of nuclear exchange, between cultured h-GF and malignant cell lines including: SAOS-2 osteosarcoma; melanoma MeIRMu, NM39, WMM175, MM200-B12; and ovarian carcinoma cells PE01, PE04 and COLO316 [9]. Although studying a range of cell lines [9], our focus was on SAOS-2 cells because we wished to contrast h-GF interactions with our previous discovery of contact dependent endothelial cell apoptosis by SAOS-2 [6]. Expression of mRNA for the inflammatory cytokine Tumour Necrosis Factor-α (TNF-αin malignant and stromal cells is associated with poor prognosis [10], [11], and fibroblasts respond to this cytokine with increased adhesion molecule expression and malignant cell binding [12], [13], hence we also investigated the effect of TNF-α and found that this cytokine significantly increased cellular sipping between h-GF and SAOS-2 [9]. In separate work, we demonstrated altered cytokine synthesis in response to TNF-α by h-GF permitted cellular sipping, compared with h-GF denied this contact dependent interaction [14].

With regard to the biological significance of cellular sipping, we observed that the morphology of neoplastic cells that have imbibed fibroblast material is intermediate to that of isolated fibroblasts and neoplastic cells cultured alone [9]. Since fibroblasts are the most prevalent non-vascular stromal cell type, we argue that uptake of fibroblast components by malignant parenchymal cells is an important source of tumour cell diversity [9], and that this may influence both tumour progression and responsiveness to anti-cancer therapies.

While cell adhesion would seem to be a minimal and essential requirement for cellular sipping, with a view to better understanding the exchange mechanism in cellular sipping it becomes interesting to consider whether or not the increased cellular sipping upon h-GF stimulation with TNF-α [9], is due to stimulation of the intercellular exchange mechanism or if it is more simply explained by increased adhesion of SAOS-2. The here described experiments characterize increased adhesion of SAOS-2 to TNF-α stimulated h-GF, and thus examine the possible role of cell adhesion as an indirect as opposed to a direct mechanism for increasing cellular sipping.

Further, Intercellular adhesion molecule 1 (ICAM-1) and Vascular cell adhesion molecule (VCAM-1) are both increased in h-GF stimulated by TNF-α [15], while increased expression of these adhesion molecules is associated with binding of malignant cells to a variety of cells and substrates [13], [16]–[18]. For this reason, the current study also examined the possible role of these two adhesion molecules in SAOS-2 binding to h-GF.

### Aims of This Study

As outlined above, we aimed to determine whether or not TNF-α increases binding of SAOS-2 to h-GF, as well as the possible role of ICAM-1 and VCAM-1 in any such binding, with a view to better understanding the role of the cytokine in cellular sipping.

## Materials and Methods

### Materials

Hank’s balanced salt solution (HBSS), cell culture medium 199 (M199), gelatine, Naphthol AS-MX phosphate, fast red violet, Tris HCl, haematoxylin and Polymyxin B solution were obtained from Sigma-Aldrich (St. Louis, USA). Iron fortified bovine calf serum was from Bovogen (Victoria, Australia). Trypsin (0.25%)/EDTA (1 mM) was from JRH Biosciences (Lenexa, USA). The antibiotics penicillin and streptomycin were from CSL Biosciences (VIC, Australia). Bovine serum albumin (BSA) fraction V and amphotericin B and were from ICN Biomedicals Inc. (Ohio, USA). Phosphate buffered saline (PBS) tablets were obtained from Oxoid (Hampshire, England), dimethyl sulfoxide (DMSO) used in freezing cells for storage was purchased from Ajax Chemicals (NSW, Australia). Anti-Human ICAM-1 antibody and FITC labelled IgG were obtained from Abcam (Cambridge, USA). Anti-Human VCAM-1 antibody was obtained from BD (Franklin Lakes, USA). Falcon FACS tubes were obtained from Becton Dickinson Labware (NJ, USA). Centrifuge tubes were purchased from Iwaki, Scitech Division (Chiba, Japan). Disposable membrane filters were obtained from Sartorius, Ministart (Gottingen, Germany). Cell culture flasks (25 cm^2^, 75 cm^2^ and 225 cm^2^) and all other cell culture plastic ware used were supplied by Costar (Cambridge, USA). TNF-α was purchased from Chemicon (Billerica, USA).

### Methods

#### Culture of fibroblasts, endothelium and SAOS-2 osteosarcoma cells

h-GF were isolated by explant culture from human gingival biopsies which were obtained with written informed consent from the Oral surgery unit, Westmead Centre for Oral Health, Westmead Hospital, under a protocol approved by the Sydney West Area Health Service Human Research Ethics Committee as outlined earlier [9], [19]. In brief, specimens were collected in sterile specimen jars containing HBSS with the antibiotics penicillin (100 U/ml), streptomycin (100 µg/ml) and amphotericin B (0.25 µg/ml). The gingival fragments were first washed with M199 containing antibiotics at least 5 times and then placed into 6 well tissue culture wells pre-scored with a scalpel to assist adhesion. Explants were cultured with a medium comprising M199 with BCS (20%) and the antibiotics at 37°C under 5% CO_2_. Medium was replaced every day for a week, after which explants were fed every 3 to 4 days with complete medium (CM) comprising M199 with antibiotics and a lower BCS concentration (10%). Explant fragments were removed by aspiration once appreciable h-GF were observed migrating onto the culture surface. Established h-GF monolayers were released with trypsin/EDTA and cultured up to 8th passage at a split ratio or 1∶3. Throughout all culture procedures and experiments, M199 contained the antibiotics penicillin (100 U/ml), streptomycin (100 µg/ml) and amphotericin B (0.25 µg/ml), while all experiments were with h-GF from 6th to 8th passage. Human umbilical vein endothelial cells (HUVEC) were obtained by collagenase perfusion and cultured in M199 supplemented with BCS (20%), Endothelial Cell Growth Supplement (50 µg/ml), heparin (30U/ml) and antibiotics as indicated for h-GF [6], [9], [20], [21].

The human osteosarcoma cell line SAOS-2 was obtained from the American Type Culture Collection (ATCC, VA, USA) and propagated at a split ratio of 1∶3 in CM at 37°C under 5% CO_2_ as outlined previously [6], [9].

#### Cell culture conditions for experiments investigating adhesion of SAOS-2 to stromal cells

Confluent h-GF were harvested with trypsin/EDTA and pelleted cells seeded at confluence into gelatinised 12 well tissue culture plates and allowed to attach overnight at 37°C under 5% CO_2_. h-GF were treated with TNF-α at concentrations of 0 nM, 0.029 nM, 0.039 nM, 0.058 nM, 0.12 nM, 0.58 nM or 1.16 nM from 0 to 24 hrs before washing twice with M199 alone and immediate evaluation of SAOS-2 binding. In some experiments, adhesion of SAOS-2 to h-GF was evaluated after an initial 24 hr period of h-GF stimulation with TNF-α (1.16 nM), and from 0 to 24 hrs of further h-GF culture either with or without continuing TNF-α stimulation at (1.16 nM).

Confluent SAOS-2 were harvested using a method identical to that for h-GF, with the exception that SAOS-2 were adjusted to a final concentration of 1.5×10^5^ cells/ml in CM before application of 1 ml/well of SAOS-2 cell suspension to washed h-GF in wells. After allowing 5 minutes for attachment, the non-adherent SAOS-2 were removed from wells by gentle washing with M199 and monolayers fixed with 10% neutral buffered formalin for 5 minutes followed by three washes with PBS and one last wash with Mili-Q water.

In some experiments, specificity of responses to TNF-α was probed by addition of Polymyxin B (1g/ml) known to bind and inactivate trace amounts of potentially contaminant lipopolysaccharide [22]–[24], or boiling TNF-α at 100°C for 30 mins [20], [21], [25]. The effect of TNF-α upon SAOS-2 with regard to subsequent SAOS-2 ability to bind h-GF was determined in additional experiments where SAOS-2 were first stimulated with TNF-α (1.16 nM) for 24 hrs prior to harvesting for adhesion assays. Some experiments were also performed substituting bovine serum albumin (BSA) to a final concentration of 4% w/v for the BCS used in adhesion assays.

#### Quantitation of SAOS-2 adhesion to stromal cell monolayers

Endogenous alkaline phosphatase activity in SAOS-2 was exploited to aid identification of these cells in adhesion assays, using a histochemical approach with Naphthol AS-MX phosphate and fast red violet as described elsewhere [9], [26], while haematoxylin was used as a convenient counterstain. Monolayers stained for alkaline phosphtase activity were observed with an Olympus CK2 phase contrast inverted microscope (Tokyo, Japan) with the phase ring removed, and photomicrographs of 4 separate fields per well recorded using a Scope photo 3.0 digital camera. Each field imaged represented a site in the mid-region between the centre and peripheral rim of an individual well, and was at a corner of what would be a square drawn within the circle of the well. The number of adherent SAOS-2 per well was determined as the total number of these cells identified in the four photomicrographs and data expressed as cells per graticule area. Average values for each treatment condition were determined across triplicate wells, while the statistical significance of individual experiments was assessed using Student’s t Test, and the results of multiple separate experiments with cells from different donors was evaluated using the Wilcoxon Ranked-Sign Test.

#### Flow cytometric (FACS) analysis of ICAM-1 and VCAM-1 expression by fibroblasts and endothelial cells

Confluent h-GF or HUVEC in gelatine coated 6 well tissue culture plates were either stimulated with TNF-α (1.16 nM) or provided with fresh CM. After from 0 to 24 hrs, cells were washed with PBS and released with trypsin/EDTA before peletting by centrifugation and resuspension in PBS with BCS (1%) and a further centrifugation. Supernatants were discarded and pellets resuspended in PBS with BCS (1%) for 20 min incubation on ice before addition of anti ICAM-1 (1 µg/ml) or VCAM-1 (5 µg/ml) and a further 30 mins incubation on ice. Cells were then washed with PBS with BCS (1%) and following centrifugation, resuspended in 100 µl of FITC labelled anti-mouse IgG (1∶100 dilution) for 30 min in ice. Following this, cells were further washed with PBS containing BCS (1%) and fixed with paraformaldehyde (2% in PBS) for 15 min at RT. Following fixation, cells were again washed with PBS containing BCS (1%), resuspended in 400 µl volumes of PBS with BCS (1%) and analysed with a Becton Dickinson LSRII Flow Cytometer using BD FACS Diva software, counting 10,000 cells per sample studied. Controls consisted of unstained cells, h-GF untreated with TNF-α, or labelled with only the secondary antibody. Data was analysed using Cyflogic 1.2.1 software and expressed graphically as fluorescence distribution profiles proportionate to the peak incidence of fluorescence.

## Results

### TNF-α Increased SAOS-2 Adhesion to Fibroblasts in a Dose Dependent Manner

**Figure 1:**
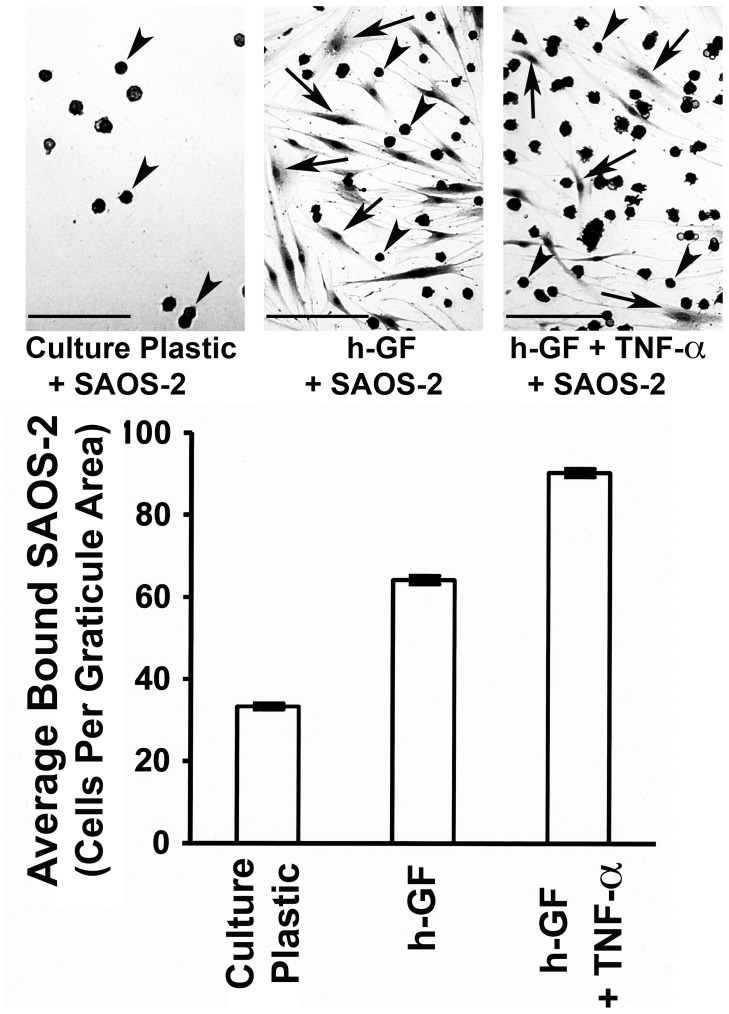
SAOS-2 binding to culture plastic and h-GF. SAOS-2 appeared as dark round cells in photomicrographs (arrow heads), as opposed to the elongated paler h-GF (arrows). h-GF increased SAOS-2 binding compared with culture plastic alone (p<0.001), and this was increased by TNF-α (1.16nM) treatment of the h-GF (p<0.001). (Bars = 50 µm).

**Figure 2:**
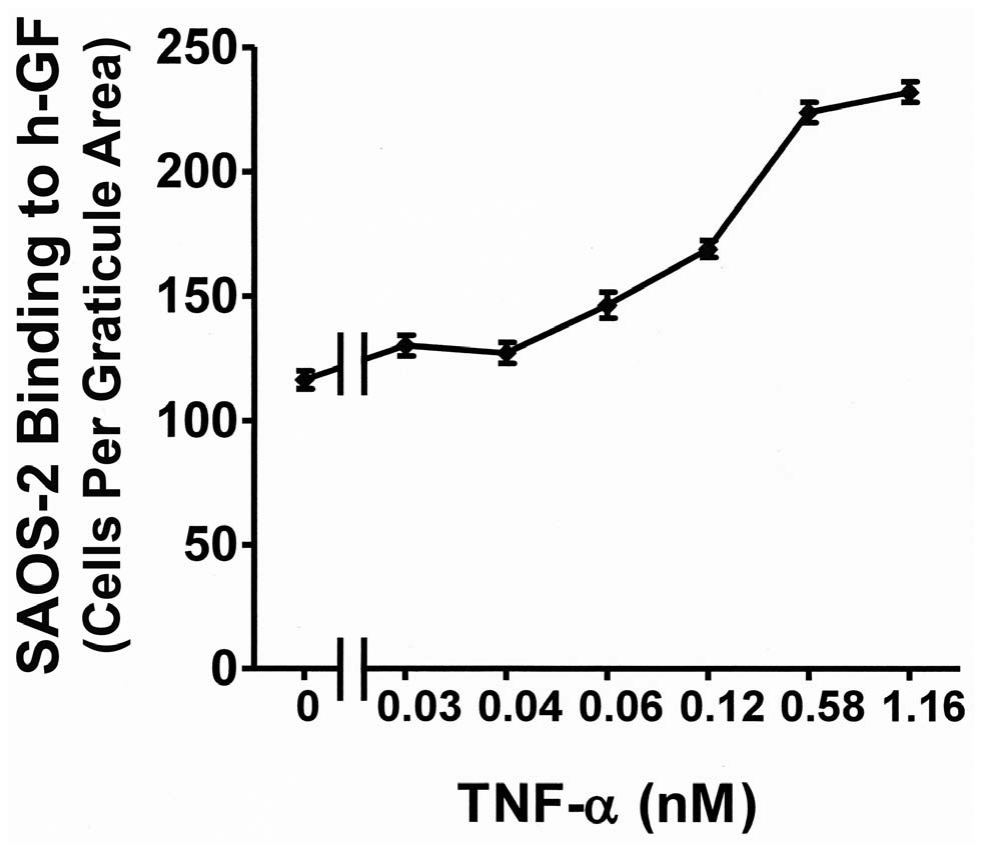
TNF-α dose response of h-GF for SAOS-2 binding. A graph shows the dose response for TNF-α of h-GF stimulated for 24 hrs with regard to subsequent SAOS-2 binding. The effect of TNF-α was seen by 0.06 nM of cytokine, and was maximal by 1.16 nM (p<0.05).

Figure 1 shows that binding of SAOS-2 to h-GF was greater than that to culture plastic alone (p<0.001), and this was increased by h-GF pre-treatment with TNF-α (p<0.001). Furthermore, increasing concentrations of TNF-α caused a dose dependent increase in the number of SAOS-2 binding h-GF with maximum activity at 1.16nM (p<0.05) and loss of activity below 0.006 nM (Figure 2). Increased SAOS-2 binding to h-GF pre-treated with TNF-α was observed in 17 separate experiments with h-GF from 6 separate donors.

**Figure 3:**
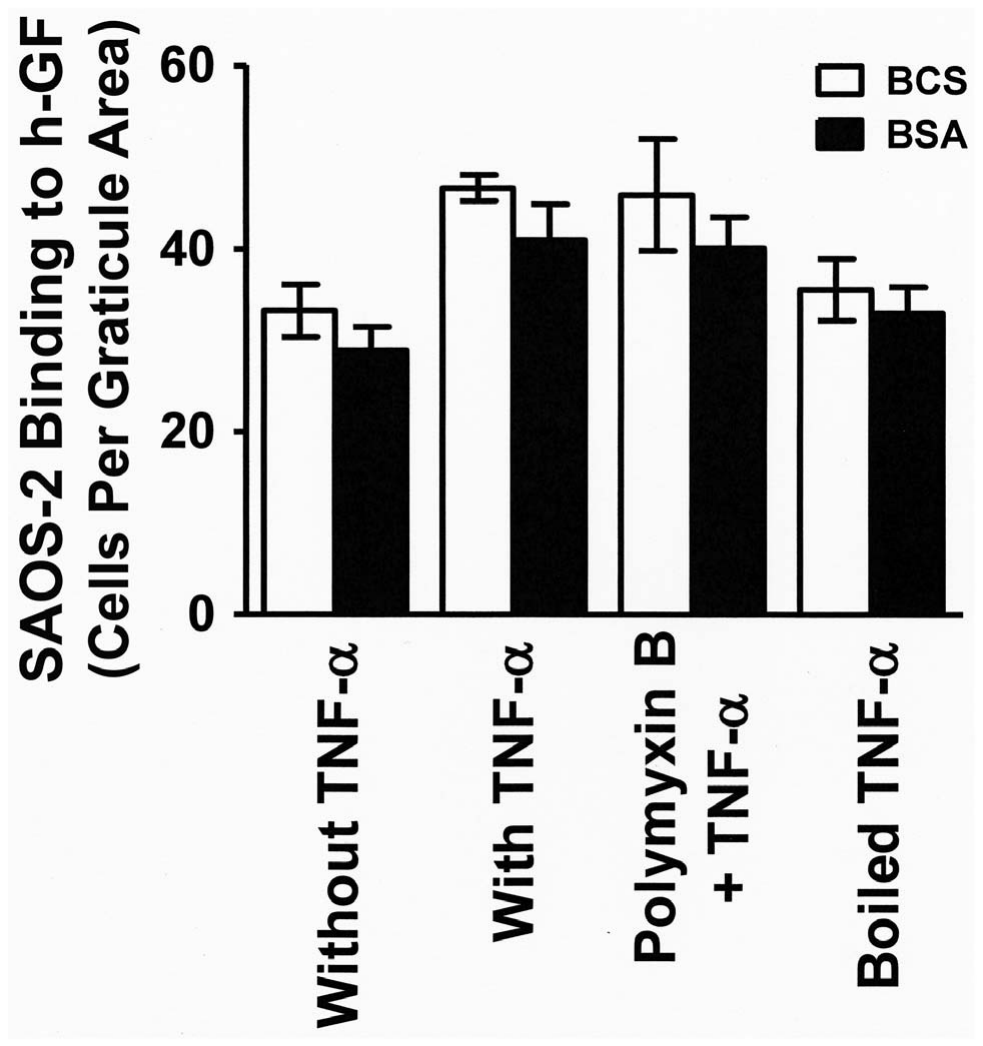
Boiling inactivated TNF-α and binding was not affected by Polymyxin B or serum. A histogram is shown illustrating SAOS-2 binding in the presence of either BCS (10%) or BSA (4%) to h-GF cultured with or without native or boiled TNF-α (0.58nM), as well as the effect of Polymyxin B (1g/ml) upon the TNF-α response. Native TNF-α significantly increased h-GF binding of SAOS-2 in the presence of either BCS (p<0.05) or BSA (4%) (p<0.05), and this effect was lost when TNF-α was boiled. A slight apparent increase in the number of SAOS-2 binding to h-GF in the presence of BCS relative to BSA was not statistically significant. The absence of an effect of Polymyxin B when included with TNF-α indicated that binding was due to TNF-α and not contaminating lipopolysaccharide.

When BCS (10%) was present in the adhesion assay there appeared to be slightly higher binding relative to when BSA (4%) was used instead, but this was not statistically significant. Polymyxin B did not affect results, while TNF-α was inactivated by boiling (Figure 3). Similar results were obtained in 2 separate experiments using h-GF from 2 different donors.

### Fibroblasts expressed ICAM-1 in a TNF-α inducible manner and blocking antibody against ICAM-1 inhibited SAOS-2 binding

**Figure 4:**
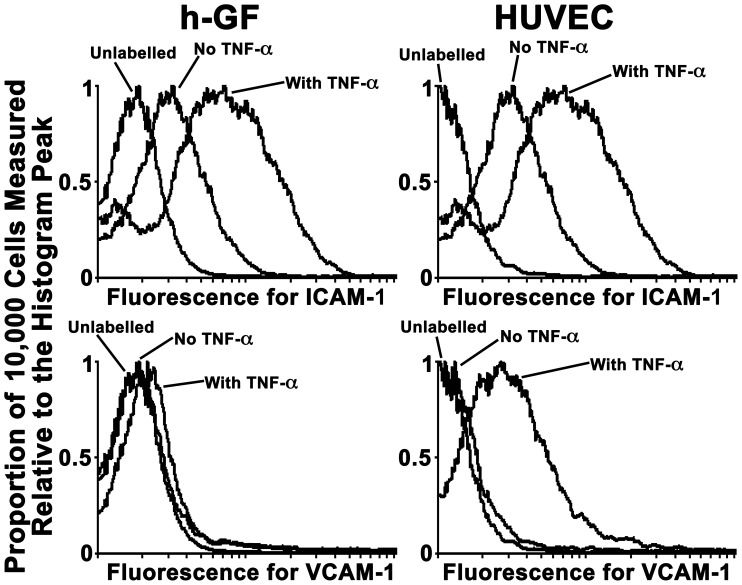
TNF-α increased ICAM-1 expression in h-GF and HUVEC, but only increased VCAM-1 in HUVEC. Fluorescence distribution profiles are shown proportionate to the peak incidence of fluorescence in each cell population studied. Background levels of ICAM-1 in both h-GF and HUVEC were appreciably increased following 24 hrs of stimulation with TNF-α (1.16nM). Although a similar effect of the cytokine was seen in HUVEC with regard to VCAM-1 expression, negligible VCAM-1 in h-GF was not significantly increased by TNF-α (1.16nM) stimulation.

**Figure 5:**
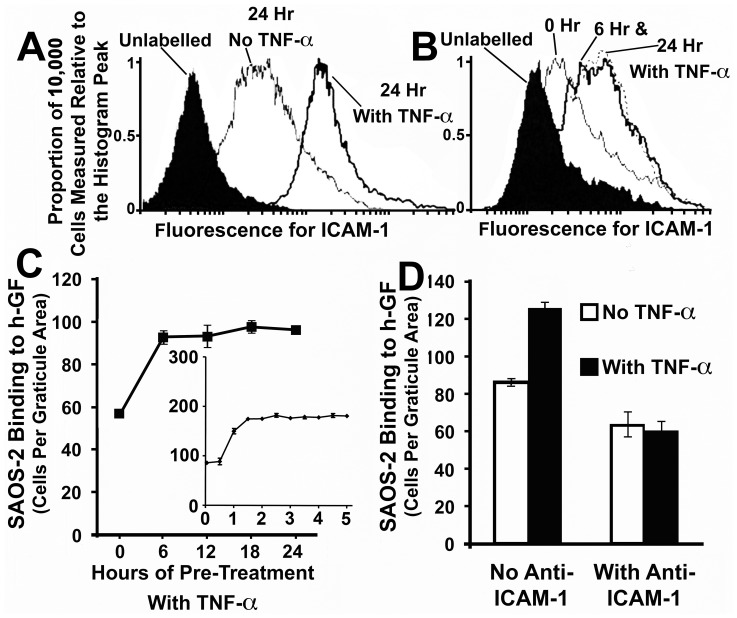
ICAM-1 was involved with TNF-α stimulated h-GF binding of SAOS-2. FACS analysis for ICAM-1 expression in h-GF with or without TNF-α (1.16nM) pre-treatment is shown (A), as well as at increasing times over 24 hr of TNF-α (1.16nM) treatment (B), compared with graphs showing the time-course of increased SAOS-2 binding by TNF-α (1.16nM) treated h-GF (C), and a histogram of the effect of blocking antibody against ICAM-1 (D). (A) h-GF expressed ICAM-1 and this was increased by TNF-α with maximal expression by 6 hr of cytokine treatment (B). (C) In an experiment performed at the same time as that shown in (5B), and using h-GF from the same donor, Maximal ICAM-1 expression correlated with maximal binding of SAOS-2 (p<0.001). As seen in the insert (C) examining the first 5 hr of cytokine stimulation in a separate time course experiment, increased SAOS-2 binding occurred between 0.5 and 1 hr of TNF-α (1.16nM) h-GF stimulation, and was maximal by 1.5 hrs (p<0.001). (D) Blocking antibody against ICAM-1 reduced binding of SAOS-2 to both untreated and TNF-α (1.16nM) stimulated h-GF (p<0.04).

FACS analysis revealed that background levels of ICAM-1 in h-GF and HUVEC were elevated by 24 hr of treatment with TNF-α (Figure 4). The cytokine had a similar effect on VCAM-1 expression in HUVEC, although but not in h-GF (Figure 4), while the absence of increased VCAM-1 expression in h-GF was seen in two further experiments with h-GF from two additional donors. Figure 5A shows similar results for h-GF ICAM-1 a separate experiment, while a time course experiment with h-GF from a different donor is also shown in Figure 5B, indicating maximal expression of ICAM-1 by 6 hrs of TNF-α treatment over a 24 hr period.

In a parallel SAOS-2 binding experiment performed at the same time and with h-GF from same donor as shown in Figure 5B, SAOS-2 binding to h-GF was also maximal by 6 hrs of TNF-α stimulation (p<0.001) (Figure 5C). The inserted graph in Figure 5C shows the result of a further separate time course experiment sampling SAOS-2 binding at progressive 30 minute intervals stimulating h-GF with TNF-α, and shows increased SAOS-2 binding after 30 minutes of h-GF stimulation, with maximal binding by 1.5 hr of cytokine stimulation (p<0.001).

To further examine the role of ICAM-1 expression in SAOS-2 binding to h-GF, three experiments were performed with h-GF from additional separate donors in which a blocking antibody against ICAM-1 was seen to reduce binding of SAOS-2 to untreated h-GF (p<0.04), while similar low levels of SAOS-2 binding were seen when blocking antibody was applied to TNF-α stimulated h-GF (p<0.005) (Figure 5D).

### TNF-α Stimulated Fibroblast Binding of SAOS-2 was Reversible Over Time

**Figure 6:**
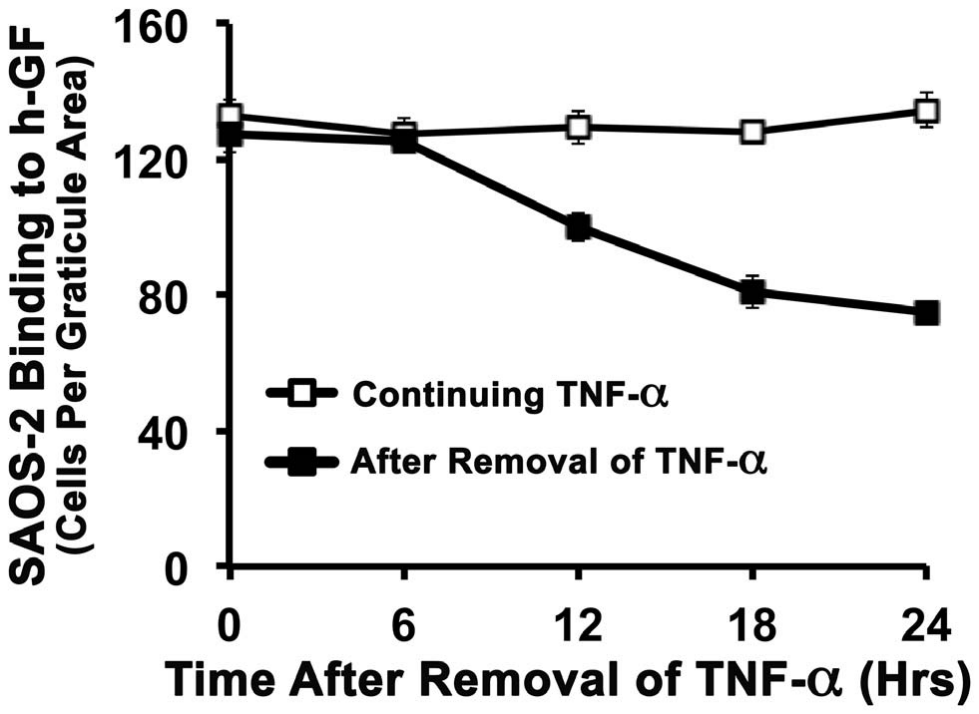
Decaying effect of TNF-α stimulation on h-GF binding of SAOS-2. A graph is shown of SAOS-2 binding to h-GF, in which h-GF were first stimulated with TNF-α (1.16nM) for 24 hrs, and then washed before further culture for from 0 to 24 hrs in M199 with BSA (4%), either with or without fresh TNF-α (1.16nM). There was decreased SAOS-2 attachment between 6 hr and 12 hrs post-cytokine stimulation (p<0.005), and this reduced to a plateau by 18 hrs (p<0.001).

To determine if TNF-α induced binding of SAOS-2 to h-GF declined after removal of the stimulus, an experiment was performed in which h-GF were first stimulated with TNF-α for 24 hrs, before being washed and further cultured with CM either with or without cytokine for increasing times, and then testing for SAOS-2 binding. Figure 6 shows that maximal SAOS-2 binding to TNF-α stimulated h-GF was retained for at least 6 hrs, but that by 12 hr some reduction in SAOS-2 binding had occurred (p<0.05), and there was further reduction to a plateau comparable to binding without cytokine stimulation by 18 hrs (p<0.001). Similar results were obtained in one separate experiment with h-GF from a different donor.

### Pre-treatment of SAOS-2 with TNF-α Reduced Binding to Fibroblasts

**Figure 7:**
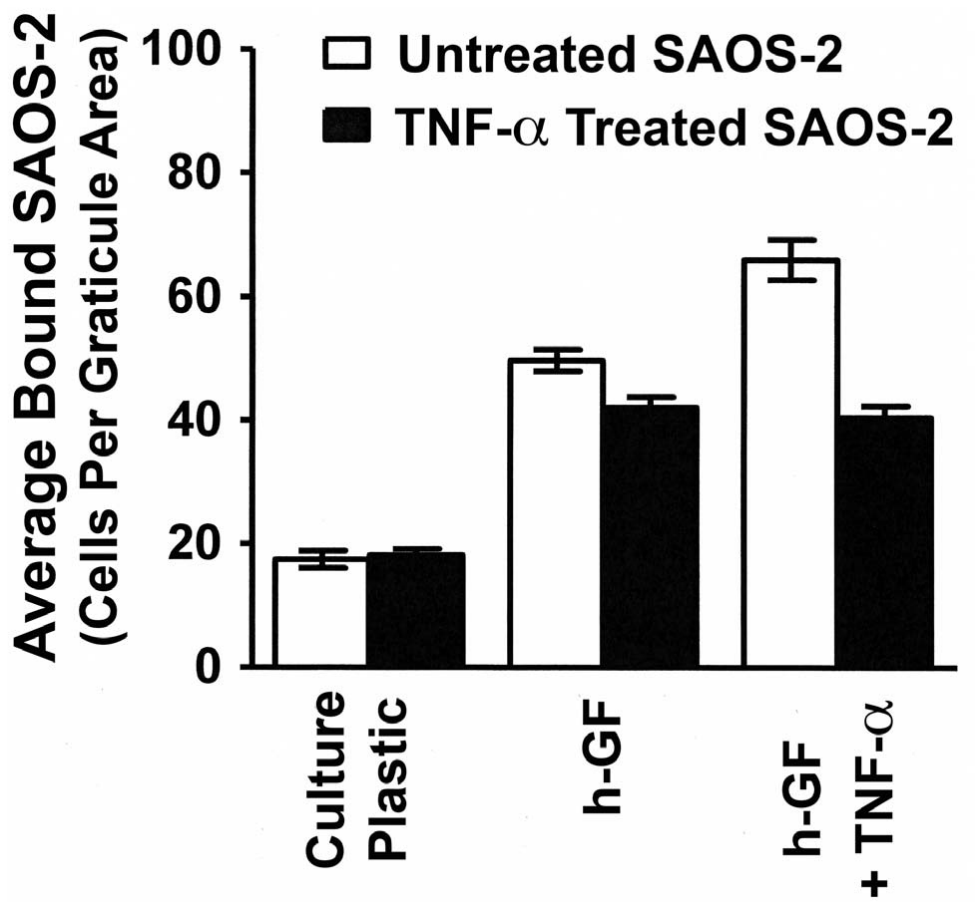
TNF-α stimulation of SAOS-2 reduced binding to fibroblasts. A histogram is shown demonstrating the effect of pre-treating SAOS-2 with TNF-α (1.16nM) for 24hrs upon subsequent SAOS-2 binding to culture plastic, untreated h-GF and h-GF previously stimulated with TNF-α (1.16nM) for 24 hrs. TNF-α treatment of SAOS-2 did not affect SAOS-2 attachment to tissue culture plastic. While the expected increased binding of unstimulated SAOS-2 to h-GF pre-treated with TNF-α was seen (p<0.002), pre-treatment of SAOS-2 with the cytokine markedly reduced binding to TNF-α treated h-GF (p<0.003), such that binding was comparable to that seen in unstimulated h-GF.

To examine a potential direct response of SAOS-2 to TNF-α with regard to h-GF binding, SAOS-2 were pre-treated with TNF-α for 24hrs, and subsequent binding to culture plastic, h-GF and h-GF pre-treated with TNF-α studied (Figure 7). Pre-treating SAOS-2 with TNF-α did not have any effect on subsequent adhesion to tissue culture plastic. Unstimulated SAOS-2 bound cytokine stimulated h-GF as expected from the above described results (p<0.002), but binding was reduced to background levels when SAOS-2 were first pre-treated with TNF-α (p<0.003) (Figure 7).

## Discussion

TNF-α increased SAOS-2 binding to h-GF, and we conclude this was mediated at least in part by increased ICAM-1 expression. Abrogation of cytokine activity by boiling supports the activity as dependent on native TNF-α conformation, and independence from Polymyxin-B is consistent with the absence of any effect by potentially contaminant lipopolysaccharide. While TNF-α and other ligands of the TNF family receptors can induce apoptosis in fibroblasts and other cells, this is typically only when protein and or mRNA synthesis are inhibited [27]–[31], while there was no sign of TNF-α induced apoptosis in either the current study or our earlier published work [9], [14].

Increased SAOS-2 binding to cytokine treated h-GF was rapid and consistent with changes in ICAM-1 expression, but nonetheless reversible with prolonged culture following removal of the TNF-α stimulus. No clear role for VCAM-1 was observed, but our observations are consistent with the work of others demonstrating increased binding of a range of cells, often via ICAM-1, following cytokine stimulation [13], [16]–[18], [32]–[35]. Although h-GF responded in a way consistent with the broader literature, the absence of significant VCAM-1 was inconsistent with an earlier report by others [15], and may reflect subtle differences in culture conditions. Detection of increased VCAM-1 in TNF-α treated HUVEC confirmed the binding integrity of the anti-VCAM-1 antibody preparation used, and hence validity of negative results in cytokine stimulated h-GF.

Had SAOS-2 binding not been increased by pre-treatment of h-GF with TNF-α, we would have concluded that earlier reported increased cellular sipping between SAOS-2 and h-GF with TNF-α [9], was likely due to cytokine activation of the exchange mechanism. However, current data are opposite to this, so that we conclude that the cytokine is unlikely to directly increase the actual mechanism for exchange between cells, but instead increases observed cellular sipping by simply increasing SAOS-2 binding to h-GF. This conclusion is further consistent with the similarity in magnitude of TNF-α stimulated cellular sipping earlier reported [9], and levels of SAOS-2 binding to cytokine stimulated h-GF in the current study.

Our data also support the idea that by increasing cell adhesion, TNF-α may encourage separate complex tumour parenchymal-stromal cell interactions, consistent with a pro-tumour activity for TNF-α noted by others [10], [11], [36], [37]. It is interesting that in the current study, the effect of TNF-α on SAOS-2 differed from that on h-GF, in that SAOS-2 treated with TNF-α had reduced binding to HDF, while similar pre-treatment of HDF with cytokine increased binding of SAOS-2 instead. This suggests that the history of exposure of individual neoplastic cells to cytokines can profoundly alter specific interactions with fibroblasts. It is tempting to further interpret this unexpected finding in terms of differential roles for TNF-α in different parts of the tumour. Where fibroblasts and neoplastic cells inhabit the same inflammatory milieu, reduced expression of adhesion molecules by neoplastic cells may counteract increased adhesion molecule expression by fibroblasts. However, where neoplastic cells invading from uninflamed tumour areas encounter local inflammation, cytokine stimulated fibroblasts may have more intense interaction with the malignant cells due to increased adhesion. While it is acknowledged that this scenario is highly speculative and would require in-vivo confirmation as well as further characterization of the SAOS-2 response to TNF-α, current data together with our separate report of altered cytokine synthesis by h-GF permitted cellular sipping [14], do highlight the extreme complexity of interactions between neoplastic and stromal cells and suggest at least partial explanation for the great morphological variability seen within different domains of single neoplasms in-vivo [38]–[40].
